# Study protocol: a mixed-methods study of women’s healthcare in the safety net after Affordable Care Act implementation – EVERYWOMAN

**DOI:** 10.1186/s12961-019-0445-y

**Published:** 2019-06-11

**Authors:** Erika Cottrell, Blair G. Darney, Miguel Marino, Anna Rose Templeton, Lorie Jacob, Megan Hoopes, Maria Rodriguez, Brigit Hatch

**Affiliations:** 1 OCHIN, Inc, 1881 SW Naito Pkwy, Portland, OR 97201 United States of America; 2 Oregon Health and Science University, 3181 S.W. Sam Jackson Park Rd., Portland, OR 97239 United States of America

**Keywords:** Women’s health, reproductive health, electronic health records, community health centres, Affordable Care Act, contraceptive care, women’s preventive care, prenatal care, postpartum care

## Abstract

**Background:**

Evidence-based reproductive care reduces morbidity and mortality for women and their children, decreases health disparities and saves money. Community health centres (CHCs) are a key point of access to reproductive and primary care services for women who are publicly insured, uninsured or unable to pay for care. Women of reproductive age (15–44 years) comprise just of a quarter (26%) of the total CHC patient population, with higher than average proportions of women of colour, women with lower income and educational status and social challenges (e.g. housing). Such factors are associated with poorer reproductive health outcomes across contraceptive, preventive and pregnancy-related services. The Affordable Care Act (ACA) prioritised reproductive health as an essential component of women’s preventive services to counter these barriers and increase women’s access to care. In 2012, the United States Supreme Court ruled ACA implementation through Medicaid expansion as optional, creating a natural experiment to measure the ACA’s impact on women’s reproductive care delivery and health outcomes.

**Methods:**

This paper describes a 5-year, mixed-methods study comparing women’s contraceptive, preventive, prenatal and postpartum care before and after ACA implementation and between Medicaid expansion and non-expansion states. Quantitative assessment will leverage electronic health record data from the ADVANCE Clinical Research Network, a network of over 130 CHCs in 24 states, to describe care and identify patient, practice and state-level factors associated with provision of recommended evidence-based care. Qualitative assessment will include patient, provider and practice level interviews to understand perceptions and utilisation of reproductive healthcare in CHC settings.

**Discussion:**

To our knowledge, this will be the first study using patient level electronic health record data from multiple states to assess the impact of ACA implementation in conjunction with other practice and policy level factors such as Title X funding or 1115 Medicaid waivers. Findings will be relevant to policy and practice, informing efforts to enhance the provision of timely, evidence-based reproductive care, improve health outcomes and reduce disparities among women. Patient, provider and practice-level interviews will serve to contextualise our findings and develop subsequent studies and interventions to support women’s healthcare provision in CHC settings.

## Contributions to the literature

- Use of electronic health record and community-level data from the ADVANCE Clinical Research Network will enable broader, more inclusive assessment of policy impacts on women’s healthcare at the patient, provider and practice levels.
- Patients in ADVANCE are evenly divided between 24 Medicaid expansion and non-expansion states; our study will provide the most comprehensive and in-depth assessment of state and local policy impacts on women’s healthcare in community health centre settings to date.
- Patient, provider and community health centre leader interviews will provide a practical context to our quantitative findings and inform subsequent studies and interventions.

## Background

Evidence-based reproductive healthcare decreases morbidity and mortality for women and their children, reduces disparities, and has lasting social and economic benefits for women, their families and their communities [1–4]. However, many women do not receive needed care, leading the Institute of Medicine to identify reproductive healthcare, including the full spectrum of contraceptive, prenatal, postpartum, and preventive education, counselling and care, as a “*critical gap*” in women’s preventive services [5]. Women who are poor and from racial and ethnic minorities are the least likely patient population to receive needed reproductive healthcare services [6]. Cost and lack of health insurance are known barriers that contribute to this gap [7]. Reproductive healthcare can result in significant out-of-pocket expenditures for women [8, 9], and even modest co-payments may deter patients from receiving needed preventive services such as mammograms and cervical cancer screening [10, 11]. Gaps in health insurance also put women at risk of not receiving needed preventive services; low-income women are disproportionately vulnerable to gaps in coverage, with approximately 40% reporting that they were uninsured at the end of 2013 [12]. However, when cost barriers are removed, women are more likely to receive needed preventive care [13]. For example, studies suggest that health insurance coverage leads to better reproductive service utilisation [14] and that women enrolled in Medicaid use preventive care at rates on par with women that have higher incomes and private insurance [12]. Moreover, for women with health insurance, lower out-of-pocket costs are associated with increased utilisation of reproductive services such as intrauterine device placement [15, 16].

Community health centres (CHCs), including Federally Qualified Health Centers (FQHCs) and similar organisations providing primary care services regardless of patients’ ability to pay, are the largest system of primary care for the United States’ low-income, uninsured and publicly insured patients as well as an important source of reproductive healthcare provision. Prior to the expansion of Medicaid under the Affordable Care Act (ACA), CHCs served one in five low-income reproductive age women in the United States [17]. Of the 20 million patients who received care in CHCs in 2011, 25% were women of reproductive age [18]. To adequately serve this population, it is imperative that CHCs provide a broad scope of reproductive healthcare services, including immunisation against HPV, screening for sexually transmitted infections, screening for cervical cancer, preconception and family planning services, and prenatal and postpartum care [19].

Historically, the federal government has relied on targeted programmes and initiatives to provide reproductive healthcare to low income women. Such efforts include extending Medicaid coverage during pregnancy and the postpartum period to millions of women not otherwise eligible for public insurance, and the Title X National Family Planning Program, which funds contraception and other sexual and reproductive health services for low-income women and men. In 2013, roughly one-quarter of CHCs reported receiving Title X funding [20] and previous work has found that Title X is the single biggest predictor of the availability of comprehensive reproductive health services in CHC settings [21, 22].

The ACA has the potential to greatly enhance the provision of women’s reproductive healthcare, especially among low-income and vulnerable populations. The ACA prioritised reproductive healthcare as an essential component of women’s preventive services [20] and expanded insurance coverage to millions of low-income Americans by providing states the opportunity to expand Medicaid coverage to adults with incomes at or below 138% of the Federal Poverty Level. In 2012, the United States Supreme Court ruled that states were not legally required to implement ACA Medicaid expansions, creating a unique natural experiment [23]. As of January 2016, 31 states and the District of Columbia had implemented Medicaid expansion and 19 had not [24], thus enabling comparison of expansion and non-expansion states. Previous studies suggested that Medicaid expansion is associated with improved healthcare access, increased CHC utilisation and improved health outcomes [25–39], but relatively fewer studies have examined the impact on reproductive healthcare [14, 19, 40, 41] and none across CHC populations in multiple states. This protocol paper describes an observational study to assess the impact of the ACA and other individual-, clinic- and state-level factors on the provision and utilisation of reproductive care in low-income and vulnerable populations served by our nation’s safety net CHCs.

### Specific aims

Reproductive Care in the Safety Net: Women’s Health after Affordable Care Act Implementation (EVERYWOMAN) is a mixed-methods study to assess differences in reproductive healthcare among women receiving care in CHCs in Medicaid expansion versus non-expansion states before and after ACA implementation. We will augment quantitative analyses leveraging electronic health record (EHR) data at clinic-, visit- and patient-levels with qualitative assessments of patient and provider perceptions of reproductive healthcare and the impact of the ACA and other contextual factors on the full spectrum of women’s healthcare provision in CHC settings. Our specific aims are as follows:

***Aim 1*:** Describe provision of women’s reproductive healthcare before and after implementation of the ACA and between Medicaid expansion and non-expansion states. ***Hypothesis:*** Provision of women’s reproductive healthcare will increase post-ACA; increases will be greater in Medicaid expansion vs. non-expansion states.

***Aim 2:*** Identify individual, clinic and state-level factors associated with provision of women’s reproductive healthcare. ***Hypothesis:*** Receipt of reproductive healthcare will be associated with (1) individual factors (e.g. age, parity, health insurance, socioeconomic status, frequency of health visits, comorbidities; (2) clinic factors (e.g. gynaecologic procedures, prenatal and obstetric care, receipt of Title X funding); (3) state-level factors (e.g. reproductive health policies, Medicaid expansion, income disparities, health education policies).

***Aim 3:*** Understand provider and patient perceptions of the provision and utilisation of reproductive healthcare in low-income and vulnerable populations in expansion and non-expansion states. ***Hypothesis:*** Patterns of reproductive healthcare utilisation and delivery are shaped by patient and provider perceptions and experiences.

## Methods

### Setting

**Fig. 1:**
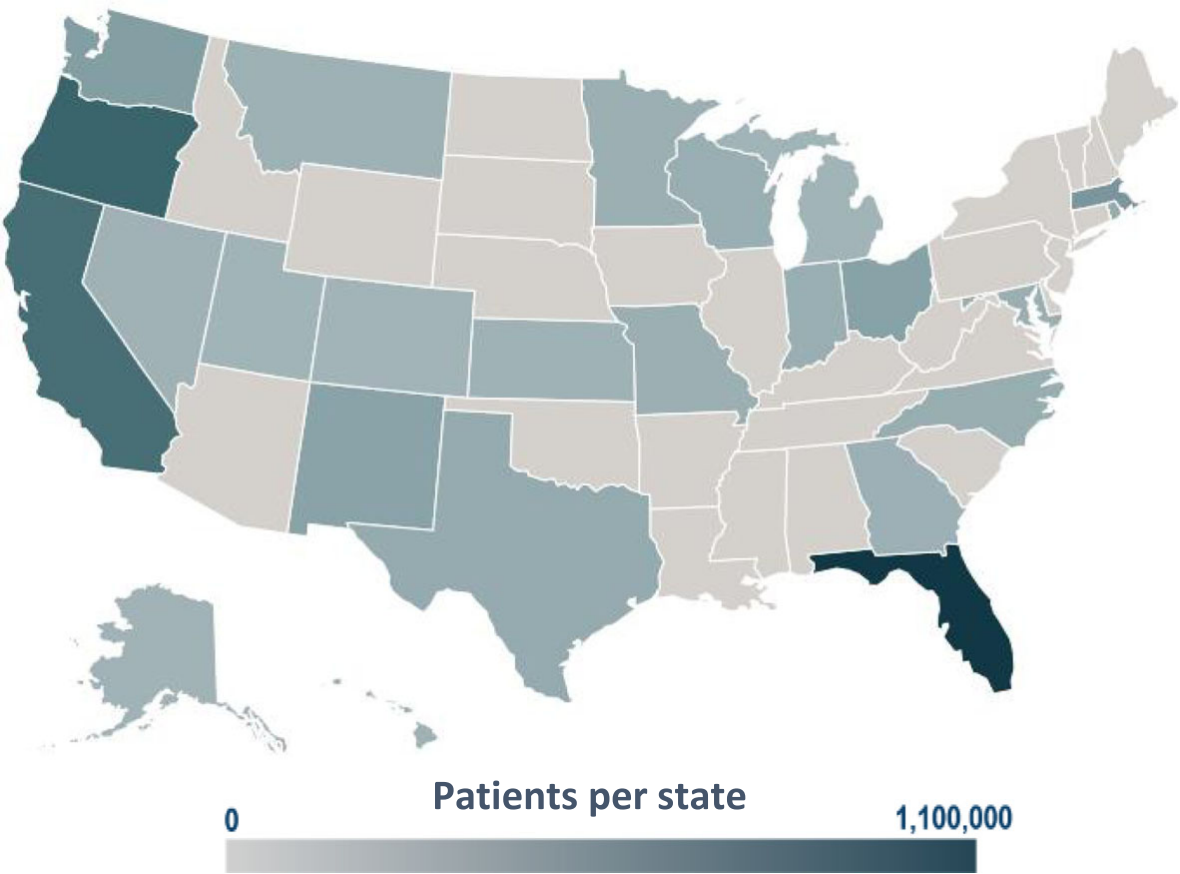
ADVANCE clinical research network patient distribution by state [42]

We will conduct this study within the ADVANCE (Accelerating Data Value across a National Community Health Center Network) Clinical Research Network, representing over 4.5 million patients from 132 CHCs across 24 states (Fig. 1) [42]. The ADVANCE collaborative is led by OCHIN, a non-profit health information and innovation company, in partnership with the Health Choice Network and Fenway Health. ADVANCE comprises patient EHR data, external clinical information (e.g. labs, imaging), demographic data specific to FQHC (e.g. income and federal poverty level), and geocoded community-level social determinants of health data (e.g. housing, education, income, employment) from publicly available sources such as the American Community Survey and United States Census, linked to individual-level clinical data using information on patient address [42, 43]. The patients in ADVANCE are in majority female (56%), with over half (53%) earning less than 100% of the Federal Poverty Level; 38% have their care covered through Medicaid and 27% are uninsured [42]. ADVANCE is one of the nation’s largest data sources for research to improve equitable health outcomes, policy and primary care delivery in underserved and vulnerable populations [42].

Qualitative components will be conducted within OCHIN’s Practice-based Research Network (PBRN) [44]. The OCHIN PBRN comprises 91 member organisations using the OCHIN-hosted Epic EHR and contributing data to ADVANCE. The OCHIN PBRN provides research-ready infrastructure and relationships to engage CHCs in qualitative work.

### Conceptual framework

**Fig. 2:**
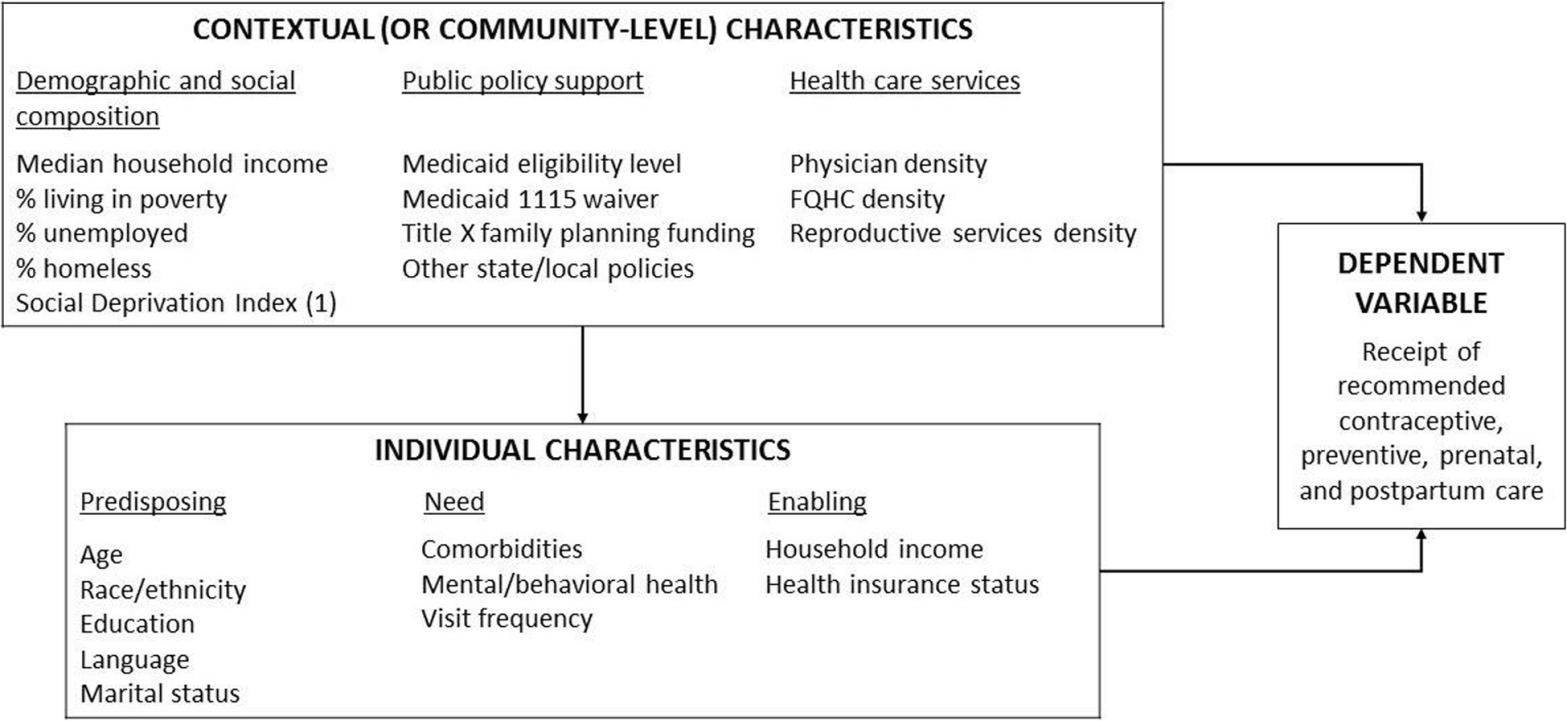
Conceptual framework adapted from Davidson et al. [45]

This study uses a mixed-methods design based on a conceptual framework for evaluating how individual and contextual variables influence receipt of needed care in low-income populations [45]. Extending on the Aday–Anderson Behavioral Health Model [46], which suggests that people’s use of healthcare services are determined by individual predisposing, enabling/hindering and need factors, Davidson et al. [45] presented a framework that includes additional contextual, or community-level, variables to represent the social, economic, political and environmental/geographic factors that influence utilisation of healthcare in low-income populations. As shown in Fig. 2 below, examples of contextual or community-level factors include the demographic and social composition of the community (e.g. median household income, percentage uninsured, percentage unemployed), public policy support (e.g. Medicaid eligibility levels, Title X funding) and availability of healthcare services (e.g. physician density, FQHC density, availability of reproductive services). At the individual level, predisposing factors include social and demographic characteristics (e.g. age, race/ethnicity, education, language, marital status) associated with decreased access to medical care; enabling/hindering factors are financial or structural factors associated with under-utilisation of healthcare (e.g. insurance status, household income); and need factors are conceptualised as an individual’s underlying health status and their frequency of engagement with the healthcare system. In our application of this framework (Fig. 2), we highlight the individual and community-level factors that may affect reproductive healthcare utilisation, conceptualised as receipt of contraceptive, preventive, prenatal and postpartum services. This schematic facilitates development of hierarchical models to assess multi-level predictors of health service delivery.

### Quantitative methods and analysis (Aims 1 and 2)

#### Sample

We will utilise patient-level clinical data from ADVANCE member clinics to describe provision of women’s reproductive services before and after ACA implementation and between expansion and non-expansion states (Aim 1) and to identify individual, clinic and state level factors associated with reproductive service provision over time (Aim 2). Our quantitative sample includes all eligible patients in the ADVANCE dataset (approximately *n* = 650,000). Patient eligibility is defined as biologically female, age 9–44 years old, and an established patient (as defined by at least two visits within the study period 2011–2016, with at least one visit pre- and one visit post-ACA implementation) at an ADVANCE member site.

#### Dependent and independent variables

Dependent variables (Table 1) represent recommended reproductive healthcare for women 9–44 years old. Dependent variables are constructed following established quality metric guidance where possible. Independent variables (Table 2) were selected and organised according to our conceptual framework and consensus guidelines [49, 52, 53]. Included variables can be feasibly measured using ADVANCE data (OCHIN has created methods to adapt preventive quality metrics for use with the EHR) [48, 54–58] and are relevant to CHC populations. The ADVANCE research data warehouse includes community-level variables from publicly available sources (e.g. American Community Survey), which are geocoded and linked to patient records using information on patient address [59]. Additional variables will be added over the course of study analysis based on review of state level reproductive health policies.

**Table 1:** Study outcomes and exclusion criteria

Outcome	Target population	Exclusions
Preventive care		
Cervical cancer screening	Females, ages 21–65	History of hysterectomy
Gonorrhoea and chlamydia screening	Females, ages 15–24	Not yet sexually active
HIV screening	Females, ages 15–65	Not yet sexually active
HPV vaccination	Females, ages 9–26	None
Contraceptive care		
Contraceptive advice, prescription, delivery (NQF #2903 & #2904) [47]	Females, ages 15–44	Sterile; Currently pregnant; Preconception visit within past 12 months
Prenatal and postpartum care		
Prenatal care	Females with completed pregnancy ‘episode’ during study period	Pregnant women not receiving prenatal care at ADVANCE member clinics
Postpartum care, including contraception (NQF #2902) [48]		
Postpartum depression screening		

*HIV* human immunodeficiency virus, *HPV* human papillomavirus, *NQF* National Quality Forum

**Table 2:** Independent variables

Variable	Description
Individual-level factors	
Age	Date of birth, age categories
Race	White, American Indian/Alaskan Native, Asian, Black, Native Hawaiian/Pacific Islander, other
Ethnicity	Hispanic/Latino
Language	English, Spanish, Russian, Vietnamese, Other
Marital status	Married, single, divorced, widowed
Household income	< 100% FPL, 100 ≤ 138% FPL, > 138% FPL
Comorbidity	Charleson Index/number of chronic conditions
Mental and behavioural health	Number of mental and behavioural health conditions
Visit frequency	Number of visits per study year
Contextual/community-level factors – Healthcare services	
Availability of healthcare services	Physician density, Federally Qualified Health Center density, availability of Planned Parenthood or Title X funded family planning clinics
Provision of gynaecologic care (clinic level)	CPT codes for colposcopy, endometrial biopsy, LEEP, and/or dilation and curettage within the study period
Provision of paediatric care (clinic level)	Care of patients under age 18
Provision of prenatal care (clinic level)	CPT codes for prenatal care during the study period
Provision of obstetric care (clinic level)	Any clinic providers performing obstetric delivery
Contextual/community-level factors – Public policy support	
Receipt of Title X funding	Clinic receipt of Title X funding during the study period
State Medicaid expansion [49]	Expansion vs. non-expansion states
1115 Medicaid Waiver	Presence/absence
Women’s and children’s health policy	Policies supporting maternal and child health
Contextual/community-level factors – Demographic and social composition^a^	
Median household income	Geocoded census tract-level median household income (American Community Survey) [50]
Percent unemployed	Percent unemployed in census tract-level median (American Community Survey) [50]
Percent living in poverty	Percent living below 100% FPL (American Community Survey) [50]
Social Deprivation Index	Composite measure of deprivation based on seven demographic characteristics collected in the American Community Survey [51]

FPL federal poverty level, CPT current procedural terminology, LEEP loop electrosurgical excision procedure

^a^Community-level demographic and social composition variables are geocoded and linked to patient clinical data in the ADVANCE research data warehouse using patient address information [43]

#### Statistical analysis

We will use descriptive statistics and data visualisation (e.g. histograms, scatter plots) to summarise baseline measures across expansion groups. Our primary methodological approach will utilise difference-in-differences (DID) methodology. DID analysis will capture the relative change in patient outcomes attributable to Medicaid expansion using non-expansion states as a reference in comparing outcomes over up to 36 months pre- and up to 36 months post-ACA implementation (Aim 1). Analysis will be extended to include individual, clinic and state-level factors that may be associated with care provision (Aim 2). This will be done through testing of three-way interactions between individual-, clinic- and state-level factors, time, and Medicaid expansion status.

Our DID approach will apply General Linear Mixed Modelling [60] to produce flexible regression models to accommodate different sources of correlation (serial, intra-clinic and intra-state), categorical and continuous covariates, and fixed and time-dependent covariates. The distribution of the outcomes of interest will be examined before selecting an analysis model; specific models will be refined through an iterative process, guided by the hypotheses, conceptual model and preliminary analyses. If we observe significant patient or clinic differences in states that did or did not expand Medicaid, we will use propensity score weighting methods to reduce the observed bias, help minimise external threats to the validity of the results and adjust for imbalances between expansion and non-expansion groups. Patient and clinic characteristics that remain unbalanced after propensity score weighting will be included in as covariates in General Linear Mixed Modelling to control for residual confounding.

### Qualitative methods and analysis

#### Sample

We will use a purposive qualitative sample to include six healthcare organisations in six states, three Medicaid expansion and three non-expansion. In the expansion and non-expansion subsets we will aim to recruit a CHC mix to include prenatal care provision, rural service area and Title X funding. We will attempt to recruit 4–6 patients, 3–5 care team members, and 1–2 health system leaders (e.g. Medical Director, Chief Operating Officer) from each participating CHC. We will conduct a total of approximately 70 interviews, 24–36 with patients and 24–42 across a mix of care providers, clinic staff and CHC leaders.

#### Data collection

We will coordinate with each site to identify and coordinate practice-level interviews with providers, staff and CHC leaders such as Practice Managers, Medical Directors or executive leaders. The study team will work closely with each site team to recruit patients from their CHC who are interested in participating in an interview. Each site will receive an impact fee of $1000 in recognition of their time coordinating and supporting qualitative activities. Interviews will be conducted by telephone and will last 45–60 min. Providers and staff will receive $25 each and patients will receive $50 each in appreciation of their time. Semi-structured interview guides are based on our conceptual framework and tailored to each respondent group. Guides will undergo iterative development over the course of the interviews. Interviews will cover perspectives and experiences with routine reproductive and sexual healthcare, including where services are accessed/delivered, perceived barriers and facilitators to care, and perceived impact of the ACA and state-level policy on care access and delivery.

#### Data analysis

Interviews will be transcribed and entered into NVivo for management of our analysis. We will follow Crabtree and Miller’s five-phase strategy [61]. Phase 1: Describing – the qualitative team will meet regularly to debrief after interviews and provide data immersion. They will provide routine, iterative summaries (crystallisation) of emerging themes for discussion with the study team. Phase 2: Organising – Themes identified in Phase 1 will be organised into a coding template. Phase 3: Connecting – In a second cycle of immersion and crystallisation, the team will review coded data to compare and connect between respondents, sites and themes. Phase 4: Corroborating, legitimising – We will seek additional data sources to confirm, refute, connect or clarify findings. Additional interpretation and review will be solicited from patient and provider representatives and other ADVANCE study teams evaluating ACA impact outcomes in CHC settings. Phase 5: Representing – Final analysis and interpretation will be reported back to target audiences and through broader study dissemination.

#### Mixed-methods integration

We will integrate qualitative and quantitative data after analysis and interpretation consistent with a mixed-method triangulation design quantitative data validation model [62]. This approach is consistent with current guidance on process evaluation of complex interventions that advocates complementing quantitative analyses with in-depth data from purposively selected samples [63].

### Limitations

#### Limitations of EHR data

EHR data have inherent limitations, but as noted above, outperform claims and self-reported data. EHR data are not originally developed for research, but our research team has conducted multiple validation studies [55, 64–67], built many EHR research datasets, and successfully conducted numerous research studies using these datasets in the past [48, 55–57, 68–71].

We anticipate missing data, either from services documented inaccessibly in the EHR (likely random) or from patients who went outside the ADVANCE network to receive services (perhaps not random). Our analyses can accommodate missing data resulting from patient attrition. We will explicitly model missingness by including related variables in the analysis as covariates [72]. If non-trivial levels of missing data are observed, we will use multiple imputation to include these patients in analyses [73]. As with any observational study, unobserved changes may occur over time, making it difficult to isolate the impact of Medicaid expansion. Our approach is strengthened by our plan to perform propensity score methods in situations where there are differences between expansion and non-expansion states, and the use of a DID approach to minimise these biases.

#### Limitations of qualitative data

Qualitative findings will be limited to a small number of clinic and state contexts due to sample size. Qualitative findings will also be limited in depth given our pragmatic, mixed-methods approach and consideration of the full spectrum of women’s reproductive care rather than a single set of outcomes (e.g. contraceptive care). We will aim to balance these limitations through our qualitative analysis by looking both within and across cases grouped by respondent roles, clinics and Medicaid expansion status and triangulating with quantitative findings [62, 74].

## Study status

This protocol is based on a proposal reviewed and funded by the Agency for Healthcare Research and Quality in 2017. At the time of submission, the research team is preparing for our first quantitative analyses and recruitment for qualitative components.

## Discussion

### The ACA has the potential to greatly enhance the provision of women’s reproductive healthcare, especially among low-income and vulnerable populations

Historically, the federal government has relied on targeted programmes and initiatives to provide reproductive healthcare to low-income women. Since the 1980s, Medicaid has provided special coverage during pregnancy and the postpartum period to millions of women not otherwise eligible for public insurance. Most states set eligibility levels for pregnant women at or near 200% of poverty [75]. Recognising the importance of reproductive healthcare prior to pregnancy, since the mid-1990s, approximately half of the states in the United States have sought and received permission from the federal government to implement Medicaid 1115 waivers to expand coverage for family planning services to individuals not otherwise eligible for Medicaid. The Title X National Family Planning Program has funded nearly 4200 family planning centres that provide high-quality and cost-effective services for low-income women and men. In 2013, roughly one-quarter of CHCs reported receiving Title X funding to provide additional resources necessary to develop the staffing and supplies needed to provide more robust family planning services [18]. Although nearly all CHCs report providing some level of reproductive healthcare, Title X funding is the single biggest predictor of the availability of comprehensive reproductive health services [21, 22], including greater access to a broad range of contraceptive services [76] such as on-site long-acting reversible contraception methods [77, 78].

Within the boundaries of this federal legislation, states have considerable latitude in how they implement the ACA, including whether to expand Medicaid, determining which preventive services are covered and whether to charge co-payments for those services. As a result, there is likely to be a great deal of variability by state with respect to the number of new women covered under the ACA and differences in both the scope and accessibility of preventive services. Perhaps most important, as a result of the United States Supreme Court ruling that states are not legally required to implement Medicaid expansions [23], as of January 2016, 31 states and the District of Columbia had implemented expansions and 19 states had not [49]. Estimates from January 2015 show significant increases in Medicaid in enrolment in expansion versus non-expansion states (26% vs. 8%) [79]. Many women directly affected by states’ decisions to expand or not expand Medicaid are seen at CHCs, thus we hypothesise that provision of reproductive healthcare services in CHCs located in expansion states will increase more post-ACA relative to their counterparts in non-expansion states.

### This study is uniquely positioned to examine receipt of reproductive health services, an essential component of women’s preventive services, at the patient level across the safety net system

Research to date has focused on the commercially insured population, clinic-level Title X data, Medicaid claims, or data from state family planning programmes. Our study allows us to see across these fragmented payors and to include the uninsured population and those who move in and out of insurance.

Moreover, alongside ACA implementation, complex state policies, social and political environments, and clinic factors will continue to shape the availability and utilisation of reproductive health services in CHCs [21, 77, 78, 80, 81]. Within these contexts, individual and community-level preferences and behaviours and differences in knowledge and attitudes about pregnancy and healthcare also contribute to patterns of reproductive healthcare utilisation and disparities in outcomes [6, 82, 83]. Ultimately, the ACA will have the biggest impact on reproductive healthcare if women not only have access to the benefits, but are also aware of the benefits and act upon them. Experts have heralded the call for research, practice and policy efforts to understand women’s preferences regarding their reproductive healthcare, how and whether CHCs are responding to their needs, and their perceptions and knowledge of the ACA, especially among low-income populations who are particularly vulnerable to disparities in access and outcome [22]. A recent *Journal of the American Medical Association* editorial stated, “…*transforming the system for women’s health is much needed; however, it is only one essential component necessary to realize change in women’s health and birth outcomes.* [Reproductive healthcare] *will continue to be a complex interplay of human behavior, social mores, technology, and science. The demand for research to explore these nuanced relationships and better understand the needs of women and families will only increase with the removal of traditional barriers to health care access*” [84]. Acknowledging the importance of these less tangible factors, in this study we augment our quantitative analysis with qualitative assessments of patient and provider perceptions of reproductive healthcare and the impact of the ACA and other contextual factors on the provision of reproductive healthcare services in CHC settings.

## Conclusion

Findings will be relevant to policy and practice, informing efforts to enhance the provision of timely, evidence-based reproductive care, improve health outcomes, and reduce disparities among low-income and vulnerable populations of women. Patient, provider and practice level interviews will serve to contextualise our findings and develop subsequent studies and interventions to support women’s healthcare provision in CHC settings.

## Abbreviations

ACA: Affordable Care Act
ADVANCE: Accelerating Data Value across a National Community Health Center Network
CHC: community health centre
DID: difference-in-differences
EHR: Electronic health record
EVERYWOMAN: Reproductive Care in the Safety Net: Women’s Health after Affordable Care Act Implementation
FQHC: Federally Qualified Health Center
PBRN: Practice-based research network
